# Molecular Genetics of Relapsed Diffuse Large B-Cell Lymphoma: Insight into Mechanisms of Therapy Resistance

**DOI:** 10.3390/cancers12123553

**Published:** 2020-11-28

**Authors:** Madeleine R. Berendsen, Wendy B. C. Stevens, Michiel van den Brand, J. Han van Krieken, Blanca Scheijen

**Affiliations:** 1 Department of Pathology, Radboud University Medical Center, 6525GA Nijmegen, The Netherlands; Madeleine.Berendsen@radboudumc.nl (M.R.B.); Michiel.vandenBrand@radboudumc.nl (M.v.d.B.); Han.vanKrieken@radboudumc.nl (J.H.v.K.); 2 Radboud Institute for Molecular Life Sciences, 6525GA Nijmegen, The Netherlands; 3 Department of Hematology, Radboud University Medical Center, 6525GA Nijmegen, The Netherlands; Wendy.Stevens@radboudumc.nl; 4 Pathology-DNA, Rijnstate Hospital, 6815AD Arnhem, The Netherlands

**Keywords:** diffuse large B-cell lymphoma, relapse, therapy resistance, mutational analysis

## Abstract

**Simple Summary:**

Many patients with the aggressive cancer diffuse large B-cell lymphoma (DLBCL) still respond poorly to treatment and suffer from relapsed or refractory disease. The identification of gene mutations that are responsible for the outgrowth of the relapsed tumor is crucial to understand the underlying mechanisms of therapy resistance. In this review, we provide a comprehensive overview of the affected genes and their biological functions in the context of therapy resistance. Furthermore, we discuss novel therapeutic strategies to treat patients with relapsed disease. We expect that the identification of these gene alterations in routine diagnostics holds great potential in guiding future therapy strategies in DLBCL.

**Abstract:**

The majority of patients with diffuse large B-cell lymphoma (DLBCL) can be treated successfully with a combination of chemotherapy and the monoclonal anti-CD20 antibody rituximab. Nonetheless, approximately one-third of the patients with DLBCL still experience relapse or refractory (R/R) disease after first-line immunochemotherapy. Whole-exome sequencing on large cohorts of primary DLBCL has revealed the mutational landscape of DLBCL, which has provided a framework to define novel prognostic subtypes in DLBCL. Several studies have investigated the genetic alterations specifically associated with R/R DLBCL, thereby uncovering molecular pathways linked to therapy resistance. Here, we summarize the current state of knowledge regarding the genetic alterations that are enriched in R/R DLBCL, and the corresponding pathways affected by these gene mutations. Furthermore, we elaborate on their potential role in mediating therapy resistance, also in connection with findings in other B-cell malignancies, and discuss alternative treatment options. Hence, this review provides a comprehensive overview on the gene lesions and molecular mechanisms underlying R/R DLBCL, which are considered valuable parameters to guide treatment.

## 1. Introduction

Diffuse large B-cell lymphoma (DLBCL) is the most common type of non-Hodgkin lymphoma (NHL) and represents a heterogenous malignancy with respect to molecular alterations, morphology, clinical behavior and treatment response [1,2]. DLBCL can arise de novo or through transformation from an indolent B-cell neoplasm [3]. Due to the aggressive character of DLBCL, this lymphoid malignancy grows rapidly, which tends to involve both nodal and extranodal sites, including stomach, testis and central nervous system [4]. Current standard first-line treatment involves several cycles of R-CHOP (rituximab, cyclophosphamide, doxorubicin, vincristine, and prednisolone) immunochemotherapy, where the addition of rituximab has led to a significantly increased survival of patients with DLBCL [5,6,7]. Despite this improvement, approximately 25–30% of patients with DLBCL still suffer from relapse and 10% show refractory disease, where outcome of relapsed/refractory (R/R) DLBCL remains poor [5,8]. Most DLBCL relapses occur within two years after diagnosis [8]. However, late relapses after 5 years still occur in a fraction of DLBCL patients, and are linked to a favorable International Prognostic Index (IPI) score, limited-stage disease and extranodal involvement at diagnosis [9,10,11,12]. Treatment of R/R DLBCL after initial therapy consists of intensified high-dose chemotherapy regimens prior to autologous stem cell transplantation (ASCT) when eligible. Alternatively, these relapsed patients may receive novel therapeutic modalities, such as cellular immunotherapy.

In order to understand the mechanisms that underly immunochemotherapy resistance in B cell malignancies, the genetic defects and biological processes associated with inferior treatment response are currently being investigated. In particular, deep sequencing of R/R DLBCL samples has provided new insight into the affected genes that contribute to the occurrence of the relapse-initiating clone. This review summarizes the current knowledge on the genetic lesions and underlying mechanisms associated with R/R DLBCL, and aims to provide an integrated framework of relevant targets for therapeutic intervention of R/R DLBCL.

## 2. Molecular Classifications of DLBCL

In an attempt to improve treatment outcome prediction and identify patients that may benefit from precision guided therapy, several molecular DLBCL classifications have been established. One of the earlier molecular classifications of DLBCL was based on gene expression profiling (GEP) related to the cell-of-origin, which resulted in the identification of two subtypes that comprise 80–85% of the cases: the activated B-cell like type (ABC) and the germinal center B-cell like type (GCB), with the remaining cases termed unclassified [13,14]. ABC/GCB classification has been further finetuned for formalin-fixed paraffin embedded (FFPE) tissue samples by other platforms, including Lymph2Cx (Nanostring) [15,16]. Patients with ABC-type DLBCL display an inferior overall survival (OS) as compared to GCB-type DLBCL. ABC subtype lymphomas frequently harbor mutations in the B-cell receptor (BCR) and the NF-kB pathway genes (*MYD88, CD79A/B, CARD11, TNFAIP3*), and display chronic active BCR signaling, whereas those of the GCB subtype commonly display *BCL2* and/or *MYC* gene rearrangements, as well as genetic lesions in *EZH2* and *PTEN* [17,18]. Despite their prognostics value, tumors within these subtypes still show heterogeneity with respect to treatment outcomes, indicating the need for more refined patient stratification strategies with improved predictive value.

As recognized by the WHO in 2016, DLBCL cases with a *MYC* translocation in combination with a *BCL2* and/or *BCL6* translocation are classified as high-grade B-cell lymphoma (HGBL) [19]. These malignancies are also known as double-hit (*MYC/BCL2* or *MYC/BCL6* translocations) and triple-hit (*MYC/BCL2/BCL6* translocations) lymphomas (DHL/THL), and represent 5–10% of DLBCL patients [20,21]. Interestingly, the majority of these lymphomas belong to the favorable GCB subtype, but show inferior outcome after R-CHOP treatment, substantiating the importance of identifying these patients. In addition, 20–35% of DLBCL cases show co-expression of MYC and BCL2 by immunohistochemistry in the absence of chromosomal translocations, which represent “double expressor” lymphomas (DEL) also associated with poor clinical outcome, but are mainly of the ABC subtype [22]. Recently, a 104-gene double-hit GEP signature (DHITsig) has been defined, which identifies an additional subgroup of GCB-type patients besides DHL/THL translocation-positive DLBCL cases, with a poor prognosis [23]. 

Whole-exome sequencing (WES), gene copy number analysis and high-throughput RNA sequencing (RNA-Seq) has resulted in novel DLBCL clusters related to treatment outcome. Schmitz et al. genetically dissected DLBCL into four subtypes: MCD (co-occurrence MYD88L265P and *CD79B* mutations), BN2 (*BCL6* fusions and *NOTCH2* mutations), N1 (*NOTCH1* mutations) and EZB (*EZH2* mutations and *BCL2* translocations), where BN2 and EZB subtypes showed better outcome [24]. Three of these genetic clusters were independently identified by Chapuy et al. (C1-BN2, C3-EZB, C5-MCD), with some discrepancy in clinical outcome for certain subtypes [25]. Wright et al. unified these two studies, defining seven genetic DLBCL clusters which correspond to different clinical outcome and potential therapeutic targeting, with about 40% of the DLBCL cases remaining unclassified [26]. In parallel, a British consortium performed targeted sequencing of 293-genes and defined five molecular subtypes [27], demonstrating partial overlap with the initial two WES studies [24,25], which includes NOTCH2-C1-BN2, BCL2-C3-EZB, and MYD88-C5-MCD, and leaving 27% of the DLBCL cases unclassified. 

## 3. Immunochemotherapy Resistance in DLBCL

First-line DLBCL treatment involves a combination of DNA damaging agents (cyclophosphamide, doxorubicin and vincristine) and the synthetic glucocorticoid prednisolone, together with rituximab. Chemotherapy resistance prototypically relates to multidrug resistance, changes in drug metabolism, inhibition of apoptosis, enhanced DNA repair and epigenetic modifications [28], while glucocorticoid resistance in lymphoid malignancies is mediated through the modulation of cell signaling, apoptosis inhibition and gene regulation [29]. It is evident that many of the biological pathways activated in B-NHL that promote the survival of the malignant B cells may also confer drug resistance, thereby inducing R/R DLBCL (see below).

The addition of rituximab to standard chemotherapy has shown beneficial effects on DLBCL treatment outcome, but also generated specific resistance mechanisms. Rituximab exerts its therapeutic effects via binding to CD20, leading to (1) complement dependent cytotoxicity (CDC), and (2) antibody-dependent cellular toxicity (ADCC) [30]. The third anti-cancer effect involves the active induction of apoptosis by inhibiting critical pro-survival pathways, including NF-kB, p38 MAPK, MEK/ERK and PI3K/AKT/mTORC1 pathways [31,32,33]. Several studies indicate that rituximab resistance mostly relates to down-regulation of CD20 expression and *MS4A1(CD20)* gene mutations in de novo tumors and R/R disease [34,35]. However, recent findings demonstrate that BCR signaling also affects the response to rituximab [36]. 

A successful approach, as shown in leukemias, to elucidate the molecular mechanisms that contribute to immunochemotherapy resistance and relapse development, involves high coverage WES analysis of tumor cell populations in both diagnosis and relapse samples. In the B-cell precursor acute lymphoblastic leukemia (BCP-ALL), *IKZF1* deletions are linked to increased relapse risk [37,38], while in chronic lymphocytic leukemia (CLL), *IKZF3* and *TP53* have been identified as relapse drivers displaying increased mutation frequency in relapsed samples after immunochemotherapy [39]. Notably, in CLL, *TP53* mutations are directly linked to chemotherapy resistance and these patients are now treated differently in order to improve treatment outcome [40,41,42,43]. These findings demonstrate the importance of identifying genetic alterations that underlie relapse and immunochemotherapy resistance in DLBCL.

## 4. Clonal Evolution of Relapsed DLBCL

The identification of relapse-associated mutations has resulted in the construction of clonal lineages from diagnosis to relapse for several cancers, including DLBCL [44,45,46,47,48]. Here, two patterns of genetic tumor evolution have been identified: (1) early divergence/branching evolution, in which the diagnosis and relapsed tumor share several variants, but mostly obtained additional unique somatic mutations, and (2) late-divergence/linear evolution, where the diagnosis and relapsed tumor share the majority of genetic alterations [45,46]. In the first scenario, already during early tumor development a subpopulation diverges, suiting a branched model that can expand into the relapsed tumor, whereas in the latter scenario, the relapsed tumor develops from a late subclone. 

As described by Juskevicius et al. [47], these models of genetic evolution correspond to two different mechanisms of resistance, namely intrinsic resistance and acquired resistance. The early divergence/branching model correlates with intrinsic resistance, where a resistant subclonal population is already present prior to treatment. After the dominant clone is eradicated due to effective therapy strategies, the subclone evolves as the relapse-initiating clone to form the relapsed tumor. In case of acquired resistance, which fits the late-divergent/linear model, resistance develops through treatment pressure, in which the subsequent genetic instability leads to genetic evolution and the acquisition of resistant mutations [49,50]. In both described scenarios, genetic variants that drive the relapsed tumor are present in a significantly higher fraction of the cancer cells at relapse compared to the primary diagnosis.

## 5. Genetic Alterations and Biological Pathways Selectively Enriched in R/R DLBCL

To identify genetic alterations that are responsible for inferior treatment outcome and relapse, several studies have investigated the genetic landscape in R/R DLBCL by targeted sequencing or WES (Table 1). Relapse-enriched and relapse-specific gene mutations have been identified by comparing variant allele frequencies (VAFs) between matched diagnosis-relapse tumor samples [34,44,45,46,48,51,52,53], or by differences in prevalence of gene mutations in samples of relapsed patients as compared to independent primary DLBCL cohorts [34,44,45,48,51,52,53]. In addition, a few studies performed mutational analysis in the diagnostic samples of patients who displayed R/R disease shortly after treatment [54,55]. Multiple studies detected genetic lesions affecting *MYC, BCL2*, *TP53* and members of the JAK-STAT signaling pathway. Other recurrent processes involve immune escape strategies and epigenetic regulation of the tumor genome. In the next paragraphs, we will elaborate on these pathways and describe their potential role in mediating therapy resistance.

### 5.1. MYC, BCL2, and BCL6 Gene Alterations

As described earlier, HGBL with gene rearrangements affecting *MYC, BCL2* and/or *BCL6*, and DEL are linked to inferior treatment response [19,20], although DEL has a better prognosis compared to DHL or THL DLBCL [19,56,57,58]. DEL are more frequent among R/R DLBCL (45%) as compared to primary DLBCL (20–35%), and associated with inferior outcome [59,60]. Additionally, DHITsig-positive cases are more common in diagnosis samples of relapsed DLBCL patients (~60%), compared to non-relapsing patients (30%) [48]. In contrast, the relative frequencies of DHL and THL in R/R DLBCL are similar to newly diagnosed DLBCL (~10%) [60,61].

#### 5.1.1. BCL2

The anti-apoptotic protein BCL2 promotes cell survival in B cells, and many BCL2 family members play a crucial role in modulating cellular stress responses [62,63]. Independent studies investigating paired diagnosis-relapse DLBCL demonstrate that several single nucleotide variants (SNVs) and copy number aberrations (CNA) in *BCL2* are enriched in relapse, while certain variants are relapse-specific [34,44,46,52,53]. Likewise, in a non-paired diagnosis and R/R DLBCL cohort studies, *BCL2* is more frequently altered in relapse, exhibiting almost doubled mutational frequencies [44,52]. Specifically, an increase in 5’UTR mutations is observed (6% vs. 17%), a region that contains various elements that control its expression [52]. Due to its anti-apoptotic properties, enhanced BCL2 expression corresponds to a poor prognosis in DLBCL [64,65].

**Figure 1:**
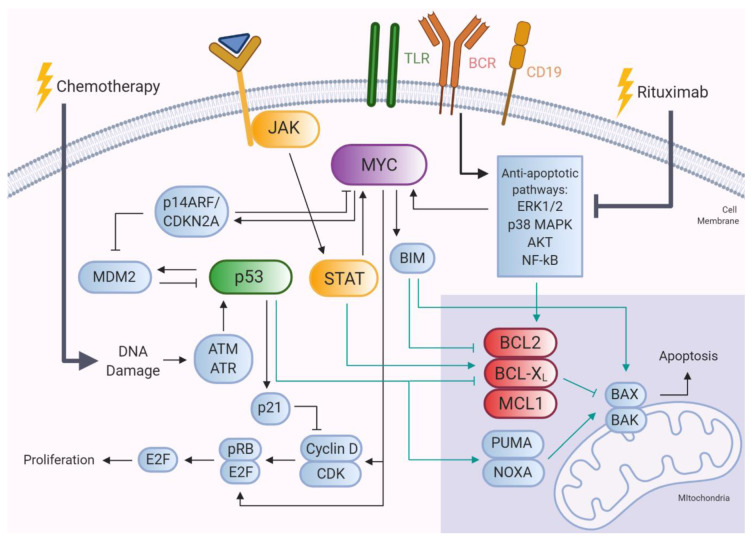
Relapse-associated genes and pathways interacting with R-CHOP immunochemotherapy. DNA damage induced by chemotherapy activates the p53 pathway, thereby inhibiting cell proliferation by p21 upregulation and inducing apoptosis through regulation of BCL2-members. Rituximab inhibits several anti-apoptotic pathways leading to downregulation of anti-apoptotic BCL2 members, and regulation of MYC. MYC, in its turn, exerts multiple functions, including regulation of the p53 pathway and pro-apoptotic executioner proteins, BAX and BAK, and the cell cycle. Activation of the JAK-STAT pathway affects proliferation and apoptosis of lymphoma cells. TLR, Toll-like receptor; BCR, B cell receptor. Turquoise lines indicate regulation of the BCL2 family members.

Mechanisms underlying its increased expression levels relate to t(14; 18)(q32; q21) IGH-*BCL2* translocation [66,67], and constitutive NF-kB activation [68]. Diagnosis DLBCL samples of patients who relapse express higher levels of BCL2 than those without relapse, which has not been observed for either MYC or BCL6 expression [45]. Furthermore, *BCL2* mutations are more prevalent in primary samples of relapsing patients than those of non-relapsing patients [45,48]. These relapse-associated SNVs targeting *BCL2* probably result in gain-of-function, thereby increasing its anti-apoptotic properties. The majority of these *BCL2* alterations most likely play a direct role in R-CHOP therapy resistance and the outgrowth of the relapse-initiating clone (Figure 1). As such, BCL2 family members are known to confer drug resistance to various chemotherapeutic agents in hematological malignancies [69,70]. Studies involving rituximab and/or CHOP resistant B-NHL cell lines reported the increased expression of anti-apoptotic BCL2 family members, including BCL2, BCL-X_L_ or MCL1, while specific inhibitors targeting these proteins abrogated these effects [71,72]. Notably, increased MCL1 expression is more frequently observed in ABC-DLBCL than the GCB subtype, which may relate to the inferior prognosis of this subtype [73]. 

#### 5.1.2. MYC

The MYC oncogene is a key player in B cell development and maturation, as it regulates the expression of multiple genes related to cell growth, differentiation, proliferation and survival [74]. Overexpression of MYC is observed in 30–50% of primary DLBCL [75,76], while missense mutations are observed in 16% of DLBCL patients [77]. *MYC* rearrangements relate to significantly inferior 5-year progression-free survival (PFS) and OS after R-CHOP treatment in patients with DLBCL [78], and are more common in diagnosis samples of primary treatment failure patients (31%) [79], although this overrepresentation is not observed in R/R DLBCL patient samples (17%) [61]. On the other hand, *MYC* mutations are more frequently observed at the time of relapse in paired diagnosis-relapse DLBCL samples, including several unique mutations that are not present at initial diagnosis [34,46,53]. Additionally, in non-paired diagnosis and R/R cohorts, *MYC* mutations are more frequently detected in relapsed DLBCL [44,51,52,53]. As such, these relapse-enriched *MYC* mutations are often located in the N-terminus and are considered to reinforce MYC’s oncogenic potential [51,80]. In addition, significant amplification of gene regions impacting *MYC* have been observed in R/R DLBCL, and not in independent DLBCL primary cohorts [52]. 

MYC primarily induces apoptosis upon overexpression, which includes the activation of the p53 pathway through the suppression of *p14ARF/CDKN2A* gene expression [81]. MYC also upregulates the pro-apoptotic protein BIM, and indirectly downregulates BCL2, BCL-X_L_ and MCL1 [82,83]. However, during lymphomagenesis, the pro-apoptotic activity of MYC is counteracted by *TP53* mutations and BCL2 overexpression. Hence, the oncogenic and chemoresistance phenotype of p53 and BCL2 is enhanced by *MYC* gene alterations [81,84]. 

#### 5.1.3. BCL6

BCL6 facilitates the proliferation of germinal center B cells after T-cell dependent antigen stimulation and represses differentiation into plasma and memory cells [85]. Distinct genetic abnormalities have been described that lead to increased BCL6 expression [86,87], and *BCL6* chromosomal translocations are present in ~25% of DLBCL [24,25]. *BCL6* gene rearrangements have been associated with poor OS in DLBCL in a meta-analysis of 22 studies, which are prognostic mainly following rituximab-containing regimens [88]. In contrast to *BCL2* and *MYC*, *BCL6* alterations other than gene rearrangements are not reported as enriched in relapsed DLBCL. Interestingly, *TP53* mutations are frequently observed in *MYC/BCL2*-rearranged lymphomas, while this is not the case for *MYC/BCL6* lymphomas [89]. As BCL6 is a known regulator of p53 [90], and p53 in turn is implicated in regulation of BCL6 [91], it could be hypothesized that for relapse formation *TP53* alterations are preferred drivers, alleviating the requirement for certain *BCL6* gene alterations.

### 5.2. TP53 Gene Alterations

Tumor suppressor p53 is involved in multiple key functions to guard the human genome against oncogenic transformation, and promotes cell cycle arrest, senescence, DNA repair and pro-apoptotic signaling under conditions of cellular stress [92,93]. *TP53* represents the most commonly mutated gene in human cancer [94], where mutant p53 evokes cell cycle dysregulation, genomic instability and uncontrolled cell proliferation. In most cases, missense mutations occur throughout the *TP53* gene, often clustering in the DNA-binding domain, which predominantly yield loss of wild-type p53 function, although gain-of-function activity has been described for several p53 mutants [95,96]. In DLBCL, *TP53* gene aberrations are detected in 35% of the patients and are mostly associated with inferior prognosis [97,98,99,100]. These include both *TP53* mutations (21%) and focal del (17p) *TP53* gene deletions (29%), which frequently co-occur resulting in bi-allelic inactivation [25,101]. In the DLBCL classification tool of Wright et al, *TP53* mutations and deletions cluster within the A53 genetic subtype with a 5-year OS of 63%, representing an intermediate risk group [26]. However, the British multicenter study showed a variable impact of *TP53* mutations on prognosis between the molecular subtypes, conferring no effect in the NOTCH2 group, but poor prognosis in the MYD88 subtype [27]. 

Most importantly, there is a significantly increased prevalence of *TP53* gene aberrations at relapse [34,44,51], which provides clear evidence that disruption of p53 function is an important driver in DLBCL relapse. Overall, mutations affecting *TP53* are clonally stable during the progression from diagnosis to relapse [34]. The increase in *TP53* mutations involves both the outgrowth of subclonal populations already present at diagnosis and the acquisition of novel relapse-specific mutations. Interestingly, ultra-deep sequencing has revealed the presence of *TP53* mutations at very low allele frequency at time of diagnosis [44]. This suggests that therapy resistance conferred by *TP53* mutations more often represents an intrinsic property of the initial tumor rather than de novo acquired [44]. Similarly, the frequency of *TP53* mutations in primary and relapsed samples of R/R DLBCL cohorts is higher than in independent primary DLBCL samples [34,44,48,51,52]. In a large cohort of R/R DLBCL (*n* = 135), *TP53* mutations were identified in 51% of the patients, whereas only 21% of primary DLBCL (*n* = 1200) showed *TP53* mutations, highlighting the importance of *TP53* alterations in relapse initiation [34]. Convergence to p53-mediated resistance in relapse samples also involves genetic alterations in p53 upstream regulators, such as the high occurrence of *CDKN2A* (*INK4A/p14ARF*) gene deletions (43% in relapse vs 15% in primary diagnosis), and mutations (30% in relapse vs 10% in primary diagnosis) [25,46,53,102,103]. 

The presence of *TP53* mutations is known to cause resistance to a wide variety of anti-cancer drugs, including alkylating agents, anthracyclines, antimetabolites, and antiestrogens [104]. The underlying mechanism is dependent on the mode of action of the drug, but for many of the cytotoxic drugs, mutant p53 interferes with the DNA damage response pathway. In p53 wild type cells, DNA damage activates protein kinases ATM and ATR, which leads to phosphorylation of p53, thereby increasing its protein stability. In this way, p53 is able to induce cell cycle arrest or apoptosis after failed cell-cycle repair through the transcriptional upregulation of cyclin dependent kinase inhibitor p21 and pro-apoptotic proteins, PUMA and NOXA, which initiate apoptosis through mitochondrial release of caspase-activating factors [43,82]. However, it is evident that p53 regulates cell survival in multiple ways, including indirect inhibition of BCL2 and MCL1 expression, and protein displacement of BCL2 family members within the mitochondria [105]. In addition, p53 impacts the immune recognition of tumor cells by regulating antigen presentation by MHC-I, as well as the expression of NKG2D ligands and PD-L1 via the transcriptional target *miR-34a* [106]. Hence, mutant p53 promotes interference with the apoptotic pathway, impairment of DNA repair and attenuated immune responses, which may lead to the acquisition of additional genetic aberrations and evasion of immune eradication. Together, this advocates an important role of *TP53* gene alterations in mediating immunochemotherapy resistance of DLBCL.

### 5.3. Mutations Targeting JAK-STAT Signaling

Activation of the Janus kinase (JAK)/signal transducer and activator of transcription (STAT) pathway occurs downstream of cytokine and growth factor receptor signaling and induces cell proliferation, differentiation and cell survival [107]. JAKs are a family of four nonreceptor tyrosine kinases (JAK1, JAK2, JAK3 and TYK2), which upon activation induce phosphorylation and nuclear translocation of STAT transcription factors, of which seven members exist (STAT1-STAT4, STAT5A, STAT5B and STAT6) [108,109]. Fine-tuning of the JAK/STAT pathway occurs at several levels, including a negative feedback loop involving proteins of the suppressor of cytokine signaling (SOCS) family. In lymphoid cells, activated STAT proteins are known to regulate the expression of critical genes involved in cell survival and proliferation, such as BCL-X_L_ and MCL1, JUN and MYC [110]. 

In DLBCL, mutations affecting the JAK/STAT pathway have been detected as relapse-enriched genetic alterations, including *JAK1, STAT6, SOCS1* and downstream target *PIM1* [34,44,48,51,53]. The role of *SOCS1* mutations in DLBCL prognosis and relapse has been more complicated to unravel, which relates to the different functions of SOCS1 [111,112], the molecular consequences of the mutations, and their impact on survival [45,113]. A recent study showed that *SOCS1* pathogenic mutations confer reduced OS in R-CHOP-treated elderly DLBCL patients [114], which fits with the notion that *SOCS1* mutations occur more frequently in relapsed DLBCL [34,51,53]. Furthermore, R/R DLBCL-enriched inactivating gene lesions target *NFKBIE* [34,51], which will lead to NF-kB-mediated production of IL-6 and IL-10 resulting in autocrine signaling and constitutive activation of JAK1/2 and STAT3 [115,116]. Collectively, these findings argue that activated JAK-STAT signaling represents a recurrent driver in relapsed DLBCL. 

### 5.4. Role of Immune Escape in Relapsed/Refractory DLBCL

**Figure 2:**
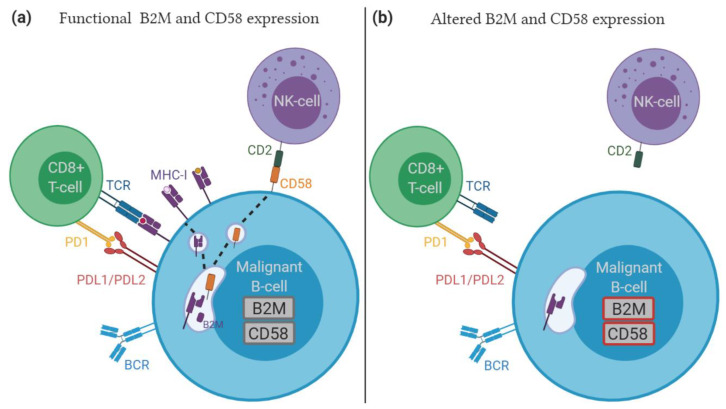
Immune escape strategies in relapsed DLBCL: (**a**) Malignant B-cell with normal B2M and CD58 expression, where B2M assembles with MHC-I, allowing antigen presentation and recognition by TCR in CD8+ T-cells. CD58 expression facilitates interaction and activation of NK- and T-cells through CD2 binding; (**b**) Malignant B-cell with inactivating B2M and CD58 mutation or expression show impaired MHC-I cell surface expression, enabling escape from immune surveillance. TCR, T cell receptor; BCR, B cell receptor; NK-cell, natural killer cell; PD-1, programmed cell death 1; PD-L1, programmed cell death 1 ligand 1; PD-L2, programmed cell death 1 ligand 2.

Evasion of immune surveillance is a critical step for DLBCL tumor development and relates to several mechanisms [117,118], some of which are associated with relapsed DLBCL (Figure 2). One immune escape strategy involves a “hide” mechanism by interfering with antigen presentation and impaired T cell recognition [119]. In DLBCL, relapse-enriched mutations have been reported in *HLA* genes [53], and in *CIITA* encoding a transactivator of MHC-II gene expression [48]. Furthermore, multiple studies have observed an increased frequency of *B2M* gene alterations or relapse-specific variants, including non-synonymous mutations, frameshift indels, and focal deletions [34,46,51,52,53]. These inactivating *B2M* gene lesions impair MHC class I folding and transport to the cell surface, causing loss of (neo-)antigen presentation and thereby allowing immune escape [120]. Alternative mechanisms that contribute to reduced MHC-I protein levels in relapsed disease involve the acquisition of gene mutations targeting (epi-)genetic regulators of MHC-I gene expression, such as EZH2 [121]. 

Attenuated B2M expression may also hold important consequences for therapies with immune checkpoint inhibitors (ICI), since proper tumor antigen presentation is required for the effective action of ICI. For instance, in melanoma, *B2M* gene alterations have been linked to ICI resistance [122,123], although in microsatellite instability-high colorectal carcinomas, patients with mutant B2M still benefited from immunotherapy [124]. With ~20% of DLBCL harboring PD-L1 positive lymphomas, the presence of *B2M* alterations may be important in ICI directed against the programmed death 1 (PD-1)/PD ligand 1 (PD-L1) interaction in R/R DLBCL [125,126]. The occurrence of PD-L1 alterations in DLBCL relates to inferior PFS after R-CHOP therapy, although in R/R patients these alterations are associated with anti-PD1 therapy response [127]. Moreover, PD-L1 alterations are linked to impaired antigen presentation and increased T-cell immune surveillance [127]. 

Interestingly, a lack of B2M and reduced MHC-I expression often co-occurs with loss of CD58 cell surface expression in primary DLBCL [128]. CD58 is a cell adhesion molecule expressed on antigen presenting cells (APCs), including B cells, that activates T cells and NK cells through binding to CD2 [129,130,131]. The absence of CD58 expression may abrogate NK cell killing of B-lymphoma cells that lack MHC-I expression (missing-self recognition), but also impairs ADCC in the context of rituximab treatment. Indeed, genetic inactivation of CD58 in DLBCL cell lines is correlated with decreased NK cell-mediated cytolysis [128] and copy number loss or mutations in *CD58* are associated with inferior prognosis in DLBCL [97]. Moreover, gain of *CD58* genetic alterations in matched diagnosis-relapse samples has been observed, including relapse-specific deletions and a frameshift indel in *CD58* gene [34,46], and *CD58* mutations are more prevalent in diagnosis samples of patients with R/R disease [48,54]. In addition, aberrant CD58 protein expression and epigenetic gene silencing has been observed in DLBCL samples without *CD58* genetic lesions, indicating alternative pathways that deregulate CD58 function [128,132]. 

### 5.5. Gene Mutations Affecting Epigenetic Regulators in Relapsed/Refractory DLBCL

Perturbation of epigenetic regulation is a common feature in DLBCL, with frequent gene alterations in histone methyltransferases (HMTs) and histone acetyltransferases (HATs) [133]. These proteins are able to regulate the modification of histones as well as the function of transcription factors, thereby controlling chromatin accessibility and affecting gene expression [134,135]. Interestingly, mutations in several epigenetic regulators, including lysine methyltransferase genes *EZH2* and *KTM2D*, as well as the HAT genes *CREBBP* and *EP300*, are clearly linked to R/R DLBCL [34,44,45,46,51,52]. 

Lymphoma-associated mutations affecting Polycomb protein EZH2 represent gain-of-function alterations, which enhance EZH2 methyltransferase activity leading to increased H3K27me3 and repression of transcription. *EZH2* mutations have been identified as a driver for relapsed DLBCL [52], with increased frequencies in matched diagnosis-relapse analysis [44,51], and independent R/R DLBCL cohorts [25,34,44]. *KMT2D* mutations, which impair KMT2D/MLL2 protein function in mediating H3K4 mono- and di-methylation, have been reported as an early event in lymphoma development [45,46]. Although allele frequencies of *KMT2D* mutations are mostly stable between diagnosis and relapse, clonal expansions have been detected in relapsed DLBCL [34,45]. As such, *KMT2D* mutations have been reported as one of the most frequently observed relapse-associated gene alterations, present in 44% of relapsed DLBCL [45]. Similarly, loss-of-function *KMT2D* mutations occur more frequently in R/R DLBCL cohorts as compared to primary DLBCL [34,44,45]. Mutations in the closely related *KMT2C* gene encoding MLL3 have also been associated with relapse [34,45,51,53], arguing that functional loss of either Trithorax-group protein fulfills an important role in the selective outgrowth of the relapse-initiating clone.

Other gene mutations that are clonally maintained in paired diagnosis-relapse samples involve the HAT genes *CREBBP* and *EP300* [34,44,46], which encode the homologous proteins CBP and p300. Moreover, gain in gene alterations and clonal expansions of *CREBBP* and *EP300* mutations have been detected [34,44,46,51,52], which are linked to glucocorticoid resistance [136]. Furthermore, *CREBBP* mutations are present at higher frequency in R/R DLBCL cohorts, compared to primary DLBCL [34,52] Notably, MLL2 and MLL3 act as coactivators at enhancers and prime enhancers for gene activation through the recruitment of CBP and p300, indicating functional interaction between these two different families of histone modifiers [137]. Other regulators of the epigenome that harbor additional SNVs at relapse include *TET2* and *BRD4* [46]. Together, these data provide strong evidence that dysregulated activity of epigenetic regulators promotes DLBCL relapse. 

An interesting observation that links these epigenetic proteins to relapse relates to their ability to functionally regulate key proteins associated with therapy resistance. KMT2D acts as a p53 coactivator as part of the ASCOM complex with ASC2, required for induction of endogenous p53 in response to DNA damage [138]. Furthermore, EZH2 has been linked to decreased CD58 expression in B-cell lymphoma, with high H3K27me3 levels at the CD58 promotor region, and increased CD58 expression upon EZH2 inhibition [132]. Indeed, *EZH2* mutations result in global H3K27me3 reprogramming that impacts B cell function and the surrounding immunological niche [139]. In addition, loss of CBP/p300 function leads to impaired acetylation of p53 and BCL6, resulting in decreased p53 activity and constitutive activation of BCL6 [140]. Thus, these epigenetic modulators may impact relapse and therapy resistance by affecting gene regulation and immune responses in both a direct and indirect manner.

## 6. Therapies Targeting Relapse-Associated Drivers

**Figure 3:**
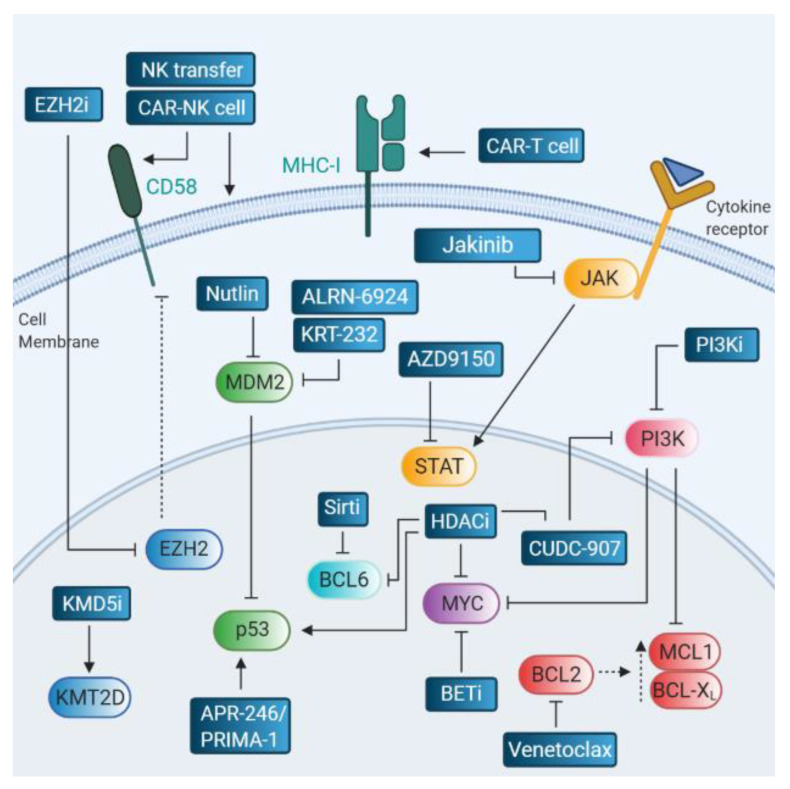
Therapeutic modalities targeting relapse-associated drivers. EZH2i, EZH2 inhibitors; NK, natural killer cell; CAR-NK cell, chimeric antigen receptor-NK cell; CAR-T cell, chimeric antigen receptor-T cell; KMD5i, KMD5 inhibitor; HDACi, histone deacetylase inhibitor; BETi, BET inhibitor; PI3Ki, PI3K inhibitor; Sirti, sirtuin inhibitor.

Multiple novel therapeutic agents are being developed to improve DLBCL treatment outcome, several of which target potential drivers of R-CHOP therapy resistance (Figure 3). In-depth discussions of specific treatment strategies for R/R B-cell lymphomas are also described elsewhere [141,142]. 

### 6.1. BCL2 Inhibitors

The BCL2 inhibitor (BCL2i) venetoclax has shown significant benefical activity in CLL and acute myeloid leukemia (AML) [143,144,145], but the therapeutic effects in DLBCL are still under investigation. The phase Ib study of venetoclax in combination with immunochemotherapy in R/R NHL patients showed CR in 7 out of 8 DEL patients [146]. The phase II CAVALLI clinical trial demonstrated 80% PFS in DLBCL patients treated with venetoclax/R-CHOP versus 67% in the R-CHOP treated cohort, and 78% PFS versus 61% in the BCL2 IHC-positive group, with no significant differences in 2-year OS [147]. The therapeutic effect of venetoclax in combination with R-CHOP for DEL patients or DA-EPOCH-R for DHL patients will be revealed in the ongoing phase III trial (NCT03984448). Possible mechanisms for venetoclax resistance is feedback upregulation of MCL1 and BCL-X_L_ expression [148], or the rare event of *PMAIP1/NOXA* gene amplification [149]. Therefore, the combination of venetoclax with strategies targeting BCL-X_L_ and MCL1, such as PI3K delta inhibitor idelalisib, seems to be promising, where synergizing effects have been observed in DLBCL cell lines and in vivo mouse models [150,151]. This also holds true for BCL2i combination with the dual-inhibitor of HDACs and PI3K, CUDC-907 (fimepinostat) [152,153], which is currently under efficiacy and safety evaluation in a phase I clinical trial in R/R DLBCL (NCT01742988). 

### 6.2. MYC Inhibitors

Given the essential role of MYC in DLBCL/HGBL pathogenesis and its association with relapse, different therapeutic strategies are being developed to target MYC-dependent lymphomas [153]. One approach involves interference with MYC-mediated transcription using inhibitors directed against BET bromodomain-containing proteins [154,155], which serve as organizers of the transcriptional machinery [156]. Phase I/II clinical trials are ongoing with different BET inhibitors, including JQ1 derivative GSK525762 (NCT01943851), and CPI-0610 (NCT01949883). Secondly, there are several strategies that target MYC protein through PI3K inhibition, which also shows therapeutic effects in the context of enhanced BCL2 activity. As such, the PI3K delta inhibitor, idelalisib, which affects MYC protein stability [157], is currently being investigated in a phase II clinical trial for R/R DLBCL (NCT03576443), although preliminary data show only limited response rates [158]. However, the dual PI3K HDAC inhibitor CUDC-907 showed promising results in a phase I study in R/R DLBCL patients harboring *MYC* translocation or amplification [159], and is currently being evaluated in a phase II clinical trial (NCT02674750). Notably, several studies have assessed the combinatorial effects of both BCL2 and MYC targeting, exploiting venetoclax with CUDC-907, BETi (NCT03255096) or PI3K inhibitor (NCT03886649, NCT04572763). Further elaboration of these synergizing effects may result in better treatment of BCL2- and MYC-positive DLBCL [152,154,160]. 

### 6.3. Targeting the p53 Pathway

There are several therapeutic strategies to target attenuated p53 activity in cancer, some of which may become beneficial for DLBCL patients harboring *TP53* mutations. One approach is to restore wild-type p53 function in tumor cells through structural reactivation of mutant p53 with small peptides, among which are PRIMA-1 and APR-246 (NCT04419389), as well as strategies targeting the MDM2/MDM4-p53 axis [161,162,163]. Nutlins are non-peptide small molecules that bind to the p53 binding groove in MDM2 hereby preventing degradation of p53 [164]. Administration of nutlin-3a in IGH-*BCL2* and *TP53*-mutant DLBCL cells, has been shown to enhance the cytotoxic effect of doxorubicin [165]. However, the activity of nutlins is compromised by other p53-pathway aberrations, such as MDM4 overexpression, and may lead to overexpression of mutant p53 [95]. A novel MDM2 inhibitor that will be in clinical trial for R/R DLBCL patients in combination with a BTK inhibitor, is KRT-232 (NCT04502394). Progress has also been made in the development of stapled peptides, such as ALRN-6924, that inhibit both MDM2-p53 and MDM4-p53 interaction, which are under (pre-)clinical evaluation for hematological malignancies (NCT02264613) [166,167]. Other candidate therapeutic approaches involve the targeting of DNA damage and cell cycle checkpoints through pharmacologic inhibition of ATM/ATR or CHK1/2 kinases, and immunotherapy with bispecific T-cell engaging (BiTE) antibody recognizing mutant p53 peptides [168].

### 6.4. Targeting the JAK/STAT Pathway

Multiple JAK inhibitors (jakinibs) have been developed, which inhibit one or multiple JAK proteins, hereby interfering with upstream JAK-STAT signaling leading to reduced cell survival [169]. Beneficial effects of these inhibitors have been observed in leukemia and lymphoma cell line and animal studies, and some strategies are currently being employed in clinical studies [170]. In R/R DLBCL a phase II trial is ongoing assessing ruxolitinib in R/R DLBCL (NCT01431209), and synergizing effects are evaluated in the combination of JAK1 inhibitor, itacitinib, with ibrutinib (NCT02760485). Additionally, the combination of itacitinib with PI3Kdelta inhibitor has been assessed in a phase I trial with R/R B-cell lymphomas [171] (NCT01905813). Although more limited than JAK inhibitors, there are some strategies to target STAT proteins, which are under clinical investigation [172]. As such, STAT3 inhibitor AZD9150 is being clinically evaluated in R/R DLBCL (NTC03527147). Other suggested strategies involve the indirect inhibition of STAT proteins through the modulation of biological STAT inhibitors, such as the SOCS and PIAS families [173].

### 6.5. Therapeutic Strategies in the Context of Immune Escape

As immune escape is observed in both diagnosis and relapsed DLBCL, it is important to develop therapy strategies that overcome this critical issue. The DLBCL associated (epi-)genetic alterations in *CD58* and *B2M* result in loss-of-function, allowing lymphoma cells to escape immune responses. For reversing epigenetic silencing of CD58 expression, EZH2 inhibitors have been shown to be effective [132]. A proposed strategy for rescuing defective B2M expression involves administration of an adenoviral vector expressing B2M, which in tumor cell lines has been shown to recover MHC-I expression, and tumor cell destruction by CD8+ T-cells [174]. However, alternative, more realistic approaches for DLBCL patients with defective antigen presentation represent cellular immunotherapies, including chimeric antigen receptor (CAR) T cell therapy directed against tumor cell surface antigens, such as CD19 and CD79 [142]. These immunotherapies are effective without the requirement of antigen presentation. At present, two different CD19-directed CAR T cell products have been approved for treatment of R/R DLBCL after two lines of systemic therapy, namely axicabtagene ciloleucel (Axi-cel; Yescarta) (NCT03391466, NCT03761056, NCT04002401), and tisagenlecleucel (CTL109; Kymriah) (NCT03642626) [175,176]. Other forms of cellular immunotherapy include adoptive transfer of NK cells and CAR NK cell targeting that could have potential for R/R DLBCL, including patients with diminished CD58 levels [177]. For ICI therapy, limited beneficial effects of nivolumab alone [178] and the combination of durvalumab with ibrutinib [179] have been reported. However, ongoing studies should reveal the efficacy of ICI- nivolumab (NCT03704714, NCT03749018), pembrolizumab (NCT03990961, NCT03401853), and durvalumab (NCT03003520)- in several combination therapies for R/R DLBCL, and establish the predictive value of *B2M* mutations in these patients.

### 6.6. Epigenetic Targeting

In the past decade, different epigenetic therapies have been explored in cancer treatment, and several HDAC targeting drugs are now FDA approved for various hematological malignancies [180]. HDAC inhibitors (HDACi) are candidate drugs to treat DLBCL patients with elevated MYC in combination with enhanced BCL2 levels, thereby deregulating MYC expression and activity [181,182,183]. HDACi also promote the accumulation of acetylated BCL6, which inhibits the ability of BCL6 to recruit co-repressors required for transcription regulation, eventually leading to cell cycle arrest and apoptosis in BCL6-positive (GCB-)DLBCL [184]. However, the clinical therapeutic efficacy of HDACi vorinostat and panobinostat have been reported to be limited in relapsed DLBCL [185,186], although HDACi abexinostat seems to be more promising [187]. Small inhibitory molecules against another class of histone deacetylases, Sirtuins, may also repress (GCB-)DLBCL growth through inhibition of BCL6 function [188]. An additional benefit of deacteylase inhibitors relates to increased levels of acetylated (wild-type) p53, which strongly stimulates its pro-apoptotic activity [189].

Specific therapeutic targeting of epigenetic modifiers has emerged for chromatin reader protein BRD4 for MYC-dependent DLBCL (BET-domain inhibitors) [154], and EZH2 for mainly GCB-type DLBCL [190]. Selective EZH2 inhibition leads to growth inhibition, differentiation and apoptosis of DLBCL cells with activating *EZH2* mutations [191,192]. The development of EZH2 inhibitor tazemetostat as monotherapy or in combination with prednisolone has been stalled (NCT01897571), but is under study in combination with atezolizumab in R/R DLBCL (NCT02220842), and R-CHOP as a first-line treatment for newly diagnosed DLBCL patients (NCT02889523). Most other genetic alterations affecting epigenetic modifiers represent loss-of-function mutations, and therapeutic targeting of these pathways is more challenging. In case of inactivating *KMT2D* gene lesions, inhibition of KMD5/JARID1 has been proposed, which is known to counteract KTM2D by demethylating H3K4me3/2, and has been linked to cancer chemoresistance [193]. Indeed, KMD5 inhibition leads to increased H3K4me3 levels showing strong anti-proliferative and cytotoxic effects in *KMT2D* mutant GCB-DLBCL cell lines with concomitant diminished BCR signaling and altered expression of BCL2 family members [194]. Thus, epigenetic targeting represents an interesting tool to counteract specific gene lesions associated with relapse and therapy resistance.

## 7. Conclusions and Future Directions

Despite the development of many novel therapeutic modalities for DLBCL treatment, R-CHOP still represents the choice of first-line therapy for most DLBCL patients. However, resistance to R-CHOP remains a persistent problem, with knowledge still limited regarding the genetic alterations that drive immunotherapy resistance, resulting in the outgrowth of the relapse-initiating clone towards a full-blown relapse. In the past years, studies investigating the mutational landscape of R/R DLBCL have improved our insight on this topic. In line with the heterogeneity of the disease, the identified genes that represent relapse-enriched mutations show variability between the different studies outside the common targets described in this review. Part of this variation can be explained by the relatively small cohorts and the different sequencing platforms in these studies. Nevertheless, many of the identified genes can be assigned to specific pathways and biological functions, thereby revealing important mechanisms that define candidate R-CHOP immunochemotherapy resistance genes. 

Not surprisingly, many of the recurrent genes are linked to the regulation of apoptosis and cell proliferation, including BCL2 anti-apoptotic family members, MYC and p53, which highlights the importance for the therapeutic targeting of these proteins and associated pathways. However, there is limited evidence for beneficial effects of targeting these pathways by single targeting agents. For this reason, combinations of targeted therapies, such as inhibitors that may target multiple anti-apoptotic BCL2 family members, combined with drugs that affect MYC, p53 pathway, immune escape genes and/or epigenetic regulators may lead to synergizing effects and could be beneficial for R/R DLBCL patients. It is evident that (epi-)genetic alterations in these candidate R-CHOP resistance drivers are not shared by all relapsed tumors, implying that there is redundancy with other gene mutations that confer therapy resistance. The identification of these genetic relapse drivers will be essential to develop targeted therapies for this group of relapse-prone DLBCL patients. Hence, more extensive NGS analysis of paired diagnosis-relapse cohorts will be required to identify the remaining class of less prevalent relapse-enriched gene aberrations. Ideally in future routine diagnostics, the presence of these genetic alterations could be used as a diagnostic biomarker for immunochemotherapy resistance, and in this way guide alternative treatment choices.

## Figures and Tables

**Table 1:** Relapse-associated genes in diffuse large B-cell lymphoma (DLBCL).

Study	Cohort Description	Cohort Size	Method	Genes Presenting R/R Enriched Variants in Paired Diagnosis-Relapse Analyses ^1^	Genes Presenting R/R Enriched Variants in Comparison with Independent Primary Cohorts ^2^
Jiang et al. 2014 [46]	Paired D-R samples	*N* = 7 (4/7 tLY)	WES	*BCL2, EP300, B2M, CD58*	
Morin et al. 2016 [51]	Paired D-R/R samples	*N* = 12 (9/12 tLY)	Targeted panel	*STAT6, EZH2, FOXO1, SOCS1, KMT2D, CD79B, NFKBIE*	
	R/R samples (taken after at least one cycle of immuno-chemotherapy)	*N* = 25	WES Targeted panel		R/R samples compared with independent primary cohort: *KMT2C, MPEG1, NFKBIZ, CCND3, STAT6, TP53, MYC, FOXO1*
Juskevicius et al. 2016 [45]	Paired D-R samples (relapse following complete remission)	*N* = 20	Targeted panel	*KMT2D, MEF2B, TET2, PRDM1,* *PTEN, EBF1*	
	Non-relapsing samples (taken at diagnosis ≥4 years relapse-free)	*N* = 20	Targeted panel		Diagnosis samples of relapsed patients compared with non-relapsing samples: *KMT2D, BCL2, PTEN, PRDM1, MCL1, CARD11*
Melchardt et al. 2016 [44]	Paired D-R/R samples	*N* = 24	Targeted panel	*TP53, RB1, EZH2*	Diagnosis samples of R/R patients compared with independent primary cohort: *NOTCH1, SMARCA4, PIM1, KMT2D* R/R samples compared with independent primary cohort: *TP53, BCL2, MYC, RB1, ATM, EZH2*
Park et al. 2016 [55]	Diagnosis samples of responsive (CR maintained > 1 year interval) vs. refractory patients (<1 year interval)	*N* = 7 responsive *N* = 6 refractory	WES		*TP53, MYD88, B2M, PRDM15, FNBP4, AHR, CEP128, BRE, SORCS3, WDFY3, CXXC4*
Mareschal et al. 2016 [54]	Diagnosis samples of R/R patients (≤1 year interval)	*N* = 14	WES		ABC: *MYD88, TBL1XR1, IRF4, CD58, PCDH17, HIST1H1B, HIST1H1C, HIST1H1D* GCB: *BCL2, DUSP2, NFKBIA, BTG2, MEF2B*
Greenawalt et al. 2017 [52]	Paired D-R/R samples	*N* = 8	WES	*CREBBP, BCL2*	
	R/R samples (after 1–8 cycles of R-CHOP)	*N* = 47			R/R samples compared with independent primary cohort: *CREBBP, BCL2, TP53, B2M,* *MYC, BTK*
Nijland et al. 2018 [53]	Paired D-R/R samples (patients that received 6–8 cycles of R-CHOP)	*N* = 6	WES	*SOCS1, PIM1, MYC, BCL2, BIRC3, BTG2, IRF4, SGK1, B2M, CALR, HLA-DR, HLA-B*	Diagnosis and relapsed samples of R/R cohort compared with independent primary cohort: *SOCS1, PIM1, MYC, HLA-DR, HLA-B*
Rushton et al. 2020 [34]	Paired D-R/R samples (tissue biopsies/ctDNA)	*N* = 57	Targeted panel	*MS4A1, KMT2D, CD79B, TBL1XR1, ZFP36L1, CARD11, BTG2, MYC, SOCS1, PIM1, TNFAIP3, MYD88, HIST1H1E, NFKBIE, TNFRSF14, BCL2, IRF4, SGK1, GNA13, B2M, FBXO11, TP53, CD58, EP300*	
	R/R ctDNA	*N* = 135			R/R samples compared with independent primary cohort: *KMT2D, TP53, CREBBP, FOXO1, NFKBIE, MS4A1*
Isaev et al. 2020 [48]	Paired D-CNS relapse samples	*N* = 5	WES	*PIM1, ETV6*	
	Diagnosis samples of systemic and CNS relapsed patients (<1 year interval) vs. non-relapsing patients (≥5 years relapse free)	*N* = 62 systemic relapse *N* = 72 CNS relapse *N* = 89 Non-relapsing	Targeted panel WES		Diagnosis samples of CNS relapse compared with non-relapsing samples: *MYD88, CD79B, PIM1* Diagnosis samples of refractory disease or systemic relapse compared with non-relapsing samples: *TP53, MYD88, BCL2, HIST1H1E, HIST1H1C, FOXO1, BTG1, CIITA, CD58, ZFP36L1*

_T_LY, Transformed lymphoma; D-R, diagnosis-relapse; R/R, relapse/refractory; CNS, central nervous system; ctDNA, circulating tumor DNA; WES, whole exome sequencing; ^1^ Genes were selected in case of gain of variant allele frequency (VAF) and/or presence of relapse-specific mutations in the R/R samples in matched DLBCL diagnosis-relapse analyses; ^2^ Genes were selected in case of higher mutational frequency in diagnosis and/or relapsed samples of R/R cohort compared with primary DLBCL cohort(s).
